# Effects of Osteopathic Visceral Treatment in Patients with Gastroesophageal Reflux: A Randomized Controlled Trial

**DOI:** 10.3390/jcm8101738

**Published:** 2019-10-19

**Authors:** Nuria Eguaras, Elena Sonsoles Rodríguez-López, Olga Lopez-Dicastillo, M. Ángeles Franco-Sierra, François Ricard, Ángel Oliva-Pascual-Vaca

**Affiliations:** 1 Escuela de Osteopatía de Madrid, 28002 Madrid, Spain; nurieguaras@yahoo.es (N.E.); f.ricard@escuelaosteopatiamadrid.com (F.R.); angeloliva@us.es (Á.O.-P.-V.); 2 Department of Physiotherapy, Universidad Camilo José Cela, 28692 Madrid, Spain; 3 Department of Health Science, Public University of Navarra, 31006 Pamplona, Spain; 4 IdiSNA, Navarra Institute for Health Research, 31008 Pamplona, Spain; 5 Instituto de Investigación Sanitaria Aragón, Universidad de Zaragoza, 50009 Zaragoza, Spain; 6 Department of Physiotherapy, Universidad de Sevilla, 41004 Sevilla, Spain

**Keywords:** GERD, gastroesophageal reflux, osteopathic medicine, pain threshold, complementary therapies

## Abstract

Osteopathic manual treatment has been recommended as a non-pharmacological therapy for Gastroesophageal Reflux Disease (GERD). However, to date, no study has supported the effectiveness of this intervention with respect to the symptoms of the disease. Our goal was to assess the effect of an osteopathic manual technique for the lower esophageal sphincter on GERD symptoms, cervical mobility and on the C4 spinous process pressure pain threshold (PPTs). Methods: A randomized, double-blind placebo-controlled trial was performed. Sixty subjects suffering from GERD participated in this study and were randomly assigned to either an experimental group (EG) (*n* = 29), who received the osteopathic technique for the lower esophageal sphincter, or to a control group (CG) (*n* = 31), who received a manual contact, which mimicked the osteopathic technique without exerting any therapeutic force. Randomization was computer-generated, with allocation concealed by sequentially numbered, opaque, sealed envelopes. The GerdQ questionnaire was used to assess symptom changes the week after intervention. Cervical Range of Motion (CROM) and algometer were used to evaluate cervical mobility and PPTs before and after both treatments. Before–after between groups comparison (*t*-test) was used for statistical analysis of the outcome, with two measurement points (GerdQ), while repeated-measures ANOVA was used for those outcomes with four measurement points (CROM and PPT). Results: The application of the osteopathic manual treatment in subjects with GERD produced a significant improvement in symptoms one week after the intervention (*p* = 0.005) with a between-groups difference of 1.49 points in GerdQ score (95% CI: 0.47–2.49). PPT C4 improved in the EG after the treatment (*p* = 0.034; η^2^ = 0.048) (between-groups difference 8.78 Newton/cm^2^; 95% CI: 0.48–17.09). CROM also increased in the EG compared to the CG (*p* < 0.001; η^2^ = 0.108) (between-groups difference 33.89 degrees; 95% CI: 15.17–52.61). Conclusions: The manual osteopathic technique produces an improvement in GERD symptoms one week after treatment, cervical mobility, and PPTs. This may mean that osteopathic treatment is useful for improving symptoms of GERD.

## 1. Introduction

Gastroesophageal reflux disease (GERD) is prevalent worldwide, and the disease burden may be increasing [1]. Prevalence varies according to country (from 2.5% in China to 51.2% in Greece) [2]. It is the most prevalent gastrointestinal disorder in the United States, and leads to substantial morbidity, though associated mortality is rare [3]. Its high prevalence has many consequences for patients, such as pain, and GERD symptoms have a negative impact on quality of life and production of work [3]. The economic burden is $9 to $10 billion per year in direct costs in the United States alone, mainly related to the use of proton pump inhibitors (PPIs) [4]. GERD is also a risk factor for developing Barrett’s esophagus and esophageal adenocarcinoma [5], which are rare in Asia, but increasing in the western population. Taking into account that GERD prevalence has been increasing in Europe and North America since 1995, it might become an even more common consultation in primary care in the near future [2,3]. 

Key factors leading to GERD are lower esophageal sphincter relaxation, crural diaphragm inhibition, esophageal shortening, and a positive pressure gradient between the stomach and the esophagogastric junction lumen. These mechanisms suggest that acid reflux events confined to the distal esophagus may produce GERD [6]. Nowadays GERD is classified as erosive or non-erosive, and typical symptoms are regurgitation and/or pirosis, but extraesophageal atypical symptoms can be found, such as cough, sibilances, wheezing, laryngitis, hoarding, sinusitis, asthma and dental erosion [7,8]. In order to diagnose GERD, pHmetry is used, along with questionnaires, and sometimes PPIs, with the aim of assessing patients’ answers. To identify GERD complications, or evaluate the need of antireflux surgery, patients undergo an upper endoscopy [7,8].

In primary care, treatment consists of hygienic–dietetic measures and PPIs. There is a suspected relationship between long-term use of PPIs and the development of polyps, mucosa degeneration and osteoporosis, so clinicians should control dosage and exposure, in particular in at-risk patients [9,10]. Novel approaches for GERD are neuromodulators, psychotherapy, hypnotherapy, cognitive and behavioral therapy [8]. 

Osteopathic consultations are mainly related to musculoskeletal issues, followed by gastrointestinal disorders [11]. World Health Organization (WHO) considers osteopathy to be a Complementary and Alternative Medicine and suggests the use of osteopathy for visceral symptoms, as well as a multidisciplinary approach to patients [12]. Moreover, visceral techniques are often used by osteopaths [13]. The effects of acupressure, Chinese spinal manipulation and osteopathy on visceral pathology have been assessed in different diseases, suggesting their potential [14,15,16,17,18,19,20]. However, only a small trial, including 30 GERD participants, showed the potential effects of manual therapy on quality of life, GERD symptomatology and PPI use [21]. Another trial showed the ability of an osteopathic technique to increase the lower esophageal sphincter pressure [22]. Two case reports also documented the clinical benefit of osteopathic management in GERD [23,24].

Gastrointestinal disease and neck pain are some of the most commonly treated acupuncture indications in the USA [25], and clinical observations suggest there might be a link between GERD, cervical pain, dystonia, and tightness through viscerosomatic reflexes by means of phrenic nerve triggering and sensitization [26,27,28,29,30]. Further, experimental gastric hyperalgesia has been shown to increase the toning of neck muscles [31,32]. In previous studies, the phrenic innervation of diaphragm and upper abdominal structures [33] has been given as the reason to treat neck pain by means of visceral osteopathic intervention in subjects suffering from digestive disorders, showing positive results in the neck area [34]. However, no previous study has analyzed the effect of visceral manual treatment in cervical sensitization and range of motion, in subjects suffering from GERD.

Accordingly, the aim of this trial was to analyze GerdQ Test changes in GERD patients after osteopathic visceral treatment, and to evaluate its effects on C4 spinous process sensitivity and on cervical range of motion.

## 2. Materials and Methods

### 2.1. Design

This study is composed of a parallel group, randomized, double-blind, placebo-controlled trial. In order to compare the effects of osteopathic manual treatment to a sham treatment, GERD symptoms, measured by a questionnaire, were set as the primary outcome, based on the previous literature [35], while C4 pressure pain threshold (PPT) and cervical mobility constituted secondary outcomes. It was registered in the Australian and New Zealand Clinical Trials Registry with registration number ACTRN 12617000188336; UTN: U1111-1181-7839.

### 2.2. Study Participants

Sixty subjects were recruited for the study by referral from a private digestive clinic in the city of Pamplona, Spain, for 5 months. Patients visited that clinic either for their first visit, for a scheduled revision, or due to a worsening of their symptoms. Those subjects who matched the selection criteria and agreed to participate were selected consecutively for their randomization.

The inclusion criteria for participants were as follows: (a) GERD diagnosis after determination of acid reflux by gastroenterologist, with upper endoscopy and/or impedance-pHmetry, which confirmed esophagitis and/or hiatal hernia; (b) aged between 18 and 70 years old and; (c) subjects who tolerate cervical movements in sitting position. Subjects were excluded on grounds of: (a) previous gastric surgery; (b) peptic ulcer; (c) previous or present gastric cancer; (d) systemic or neurologic diseases; (e) pregnancy; (f) recent fractures or cervical trauma; (g) patients receiving chemotherapy or radiation therapy, or; (h) mental disorders which might affect the obtained data.

### 2.3. Randomization, Blinding and Allocation

Randomization was undertaken using a computerized randomization system (randomized.com), and allocation concealment was guaranteed by sequentially numbered, opaque, sealed envelopes. An outside coworker safeguarded the sequence for those participating in the study. Evaluators who collected or analyzed data remained unaware of the aims of the study as well as the treatment allocation group, to ensure participant blinding and outcome assessor blinding, respectively [36]. The practitioner did not take part in symptom evaluation or outcome measurement.

### 2.4. Study Protocol

Since participants were recruited in a digestive clinic, they might not be used to manual interventions for GERD management, so, in order to avoid a high level of withdrawal, they received only two sessions, with a weeklong time lapse between the first and second. During each session, measures of outcomes were taken before and after the experimental or control intervention. The same procedure was performed on all subjects, and no measures were taken to encourage or discourage intake of medication.

**Figure 1:**
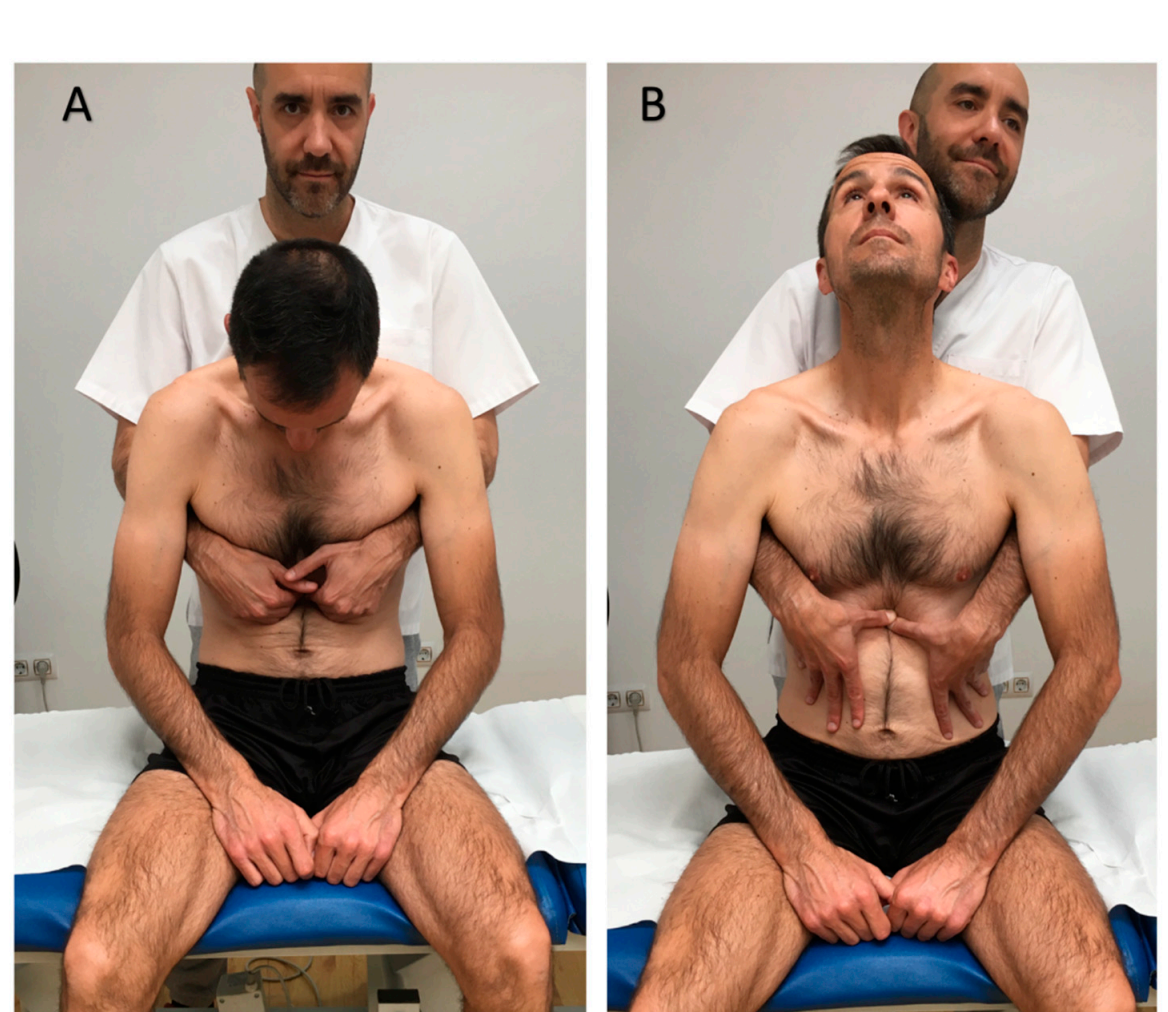
Osteopathic manual technique for the lower esophageal sphincter. (**A**). Initial position. (**B**). Final position.

Experimental group (EG) went under a visceral osteopathic technique, which is commonly used for GERD [23]. To perform the technique, the patient was seated, and the osteopath stayed behind, with the osteopath´s hands placed in the patient´s epigastric area. The patient was then asked to bend in flexion while breathing in, so the osteopath deepened her hands on the patient´s epigastrium. After that, the patient was asked to straighten his whole spine and to extend his neck while breathing out, and at that time the osteopath pushed caudally with her hands. The procedure was repeated for 5 min. This technique has been proposed for patients suffering from GERD [37] (Figure 1).

The sham technique in control group (CG) was performed by the same investigator. In this case, the investigator maintained her hands in contact with the patient’s ribs. The patient had to reproduce the same deep breathing as the intervention group for 5 min; however, the investigator’s hands only maintained physical contact with the patient, without exerting any pressure, or putting any incentive or restriction on the tissues or the movements of the thoracic cage.

### 2.5. Primary Outcome: GERDQ Test

Changes in GerdQ test punctuation were analyzed. The GerdQ test consists of six items of gastroesophageal reflux symptoms in the previous 7 days, and it is recognized as a validated method to assess gastroesophageal reflux symptoms [7,38,39,40]. This questionnaire has sensitivity, specificity, positive and negative predictive values of 72%, 72%, 87% and 50%, an internal consistency by the Cronbach´s alpha coefficient of 0.93, and very good reproducibility [41,42]. Subjects filled out the questionnaire before the first intervention and again the week before the application of the second intervention.

### 2.6. Secondary Outcome: PPT and Cervical Mobility

Both outcomes were measured after a two week training period, by a nurse with 10 years of experience.

Pressure pain threshold (PPT): pressure was applied on the spinous processes of the fourth cervical vertebrae. PPT measurements were made with JTECH Commander algometer (J-Tech Medical Industries, Midvale, UT, USA) [43]. Algometry has proved to be a reliable instrument for measuring PPT. Intrarater reliability has proved to be almost perfect (ICC = 0.94–0.97), interrater reliability substantial to near perfect (ICC = 0.79–0.90), and test–retest reliability substantial (ICC = 0.76–0.79) [44]. Pressure was uniformly increased over C4, and all patients were given the identical instruction, “let me know when the sensation of pressure becomes uncomfortable or painful” [45]. At this point, the pressure was immediately released, and the plunger was retracted by the evaluator. Three measurements, with a resting period of 30 seconds between each measurement, were made for each evaluation, and the mean of the three measurements was taken as the reference value [46]. Measurements were taken before and after the first and second intervention (measured in Newton/cm^2^).

Cervical mobility: Cervical mobility was measured using the Cervical Range of Motion (CROM-device^®^) tool (Performance Attainment Associates, St. Paul, MN, USA), which is a floating compass attached to the apex of the head by velcro straps. An intratester reliability, in the range 0.87–0.96, has been reported for this device, with a standard error of measurement between 2.3° and 4.1° [47]. Further, its validity has been confirmed compared to the FASTRAK motion analysis system (Polhemus, Colchester, VT, country, UK), showing a between-day reliability in the range 0.89–0.98, with standard error of measurements for the six cervical movements between 1.6° to 2.8° [48]. Patients kept seated, and intervention and measurements always took place in the evening [41]. CROM data were recruited before and after the first and second interventions, with one week between them. Active movements analyzed were flexion, extension, side bending and rotation to both sides [49,50,51]. Three measurements were made for every movement during each evaluation, and the mean of the three measurements was taken as the reference value. Arithmetic sum was calculated for each range of motion and this variable was called “cervical mobility”.

Further, patients were asked in the second visit about any complaints, events or reactions during the week.

### 2.7. Statistical Analysis

Statistical analyses were performed using SPSS 22.0 software (SPSS Science, Chicago, United States). We have presented descriptive statistics in tables as both mean, standard deviation and 95% confidence interval (CI) for continuous measures, or percentages for categorical responses. The two-sample *t*-test, or the X^2^ test, was used to examine potential differences in baseline values and demographic variables between the two groups. We examined the normality (Shapiro–Wilk test) of outcome variables and found that body mass index, PPT C4 and the GerdQ test were not normally distributed; hence, we applied U Mann Whitney to analyze baseline differences between the two groups. With respect to the GerdQ test, which was measured only twice, pre–post improvement was calculated, and *t*-test was used to analyze the intergroup comparison. To avoid the influence of eventual baseline imbalance, the between-groups difference for the GerdQ test was also analyzed by ANCOVA, using baseline values as a covariable, and R^2^ was then used as a measurement of the effect size. Repeated measures analysis of variance, with linear mixed effects model, was used to test the profile of the change in PPT C4 and cervical mobility, since these outcomes were measured four times. Effect size (η^2^) was calculated to find differences between intervention and sham groups. Effect sizes, both η^2^ and R^2^, were categorized as small (<0.01), medium (0.01–0.06), or large (>0.14). Absolute between-groups differences were also calculated. Bivariate correlations between the outcome variables were analyzed using the Pearson coefficient, and the influence of baseline GerdQ on symptoms’ improvement was similarly studied. Significance level was set at α = 0.05.

Sample size was estimated for the GerdQ test with Granmo v7.12 (IMIM Hospital del Mar, Barcelona, Spain). Accepting an alpha risk of 0.05 and a beta risk of 0.2 in a bilateral contrast, 29 subjects are needed in every group to detect a difference equal or superior to 1.5 units. It is assumed that standard deviation is 1.92 units. Loss of follow-up was estimated in 10%.

### 2.8. Ethical Considerations and Data Protection

The study was conducted in accordance with the ethical standards of the Declaration of Helsinki [52], and the confidentiality of patient data was respected [53]. This study received ethical approval by the Ethical Research Committee of the Camilo José Cela University (Spain, ITCPERG). Before their participation, patients were given written information with regard to the objectives and procedures of the study, and agreed to participate by signing a statement of informed consent.

## 3. Results

### 3.1. Sample

**Figure 2:**
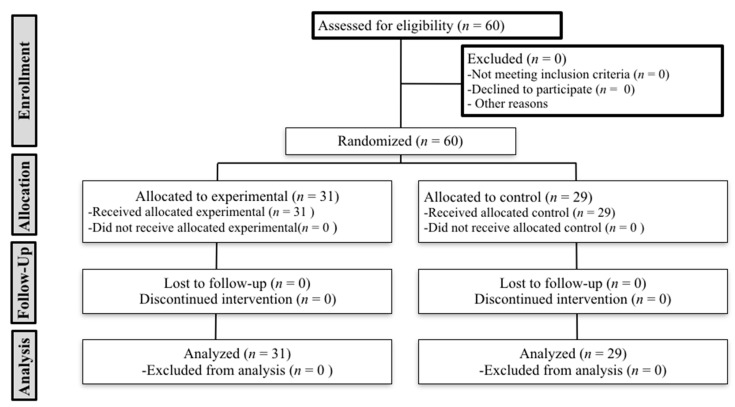
CONSORT Flow Diagram.

Figure 2 shows details of recruitment. A total sample of 60 patients were included in the study. Thirty-one patients were randomized to the EG and 29 to the CG. Twenty-nine patients were men (15 men (48.4%) in the EG, versus 14 (48.3%) in the CG) and 31 were women (16 women (51.6%) in the EG, versus 15 (51.7%) in the CG). Patients were aged between 20 and 70 years (48.80 ± 13.80 years). The 36.7% of subjects were receiving medical treatment with proton pump inhibitors. The total sample had 4.48 ± 3.46 points on Symptoms of GerdQ test (3.79 in the CG, versus 5.13 in the EG). We found no significant differences in sample characteristics between groups. Table 1 shows the characteristics of the sample.

### 3.2. Outcome Variables

The results are shown in Table 2. The scores of the GerdQ test decreased in the EG, compared to those in the control group. The application of the osteopathic manual treatment in subjects with GERD produced a significant improvement in symptoms one week after the intervention, compared to the application of the sham maneuver (M = 0.448 ± 1.84, t(58) = 2.94, *p* = 0.005) with a between-groups difference of improvement of 1.49 points in their GerdQ score (95% CI: 0.47–2.49) (Table 3). ANCOVA analysis confirmed these results, showing a medium effect size (F(2,57) = 6.126, *p* = 0.016; adjusted R^2^ = 0.671). With respect to PPT C4, a significant time by-group interaction effect was found (F (3,174) = 2.94, *p* = 0.034, η^2^ = 0.048), showing an improvement in the evolution of the EG, with higher PPT values after the treatment. Finally cervical mobility increased in the EG compared to the CG, with a significant time by-group interaction effect (F (3,174) = 7.049, *p* < 0.001, η^2^ = 0.108).

On the other hand, a correlation was found between baseline values of the questionnaire and GerdQ improvement (r = −0.322; *p* = 0.044), showing that improvement is lower in subjects with stronger symptoms. We have identified a significant correlation between baseline symptoms of GerdQ test and C4 PPT, showing that subjects with greater gastroesophageal reflux symptoms presented lower PPT in C4 spinous process (r = −0.317; *p* = 0.014). No other correlation was found (*p* > 0.05 in all cases).

No patient in the CG referred to any adverse event or complaint. However, two patients in the EG explained that they had felt hypersensitivity in the epigastric area where the osteopath had placed her hands to perform the technique. No other kind of adverse event was reported. In the CG, eight participants (27.58%) got worse in the second evaluation of GerdQ, while this only happened in one subject (3.22%) in the EG.

## 4. Discussion

The main objective of this research was to evaluate the effects of osteopathic visceral treatment on GERD symptoms. Our results show that GerdQ test score improves during the week after the intervention. Secondary outcomes in this study were to evaluate C4 spinous pressure sensitivity and cervical range of motion after the osteopathic technique application. Our results state that range of motion and PPT on C4 increase, especially after the second intervention. We have also found that the worse the reflux symptoms (GerdQ test score), the lower PPT in C4. Moreover, higher reflux symptoms are also related to lower GerdQ improvement, obtained after the application of this osteopathic technique for only a 5 min session.

To our knowledge, our study is the first non-preliminary randomized controlled trial which shows the effect of manual intervention in patients suffering from GERD. Besides, it points out not only the effect on visceral symptoms, but also on somatic manifestations, although no data were collected about PPI consumption decrease. The main limitation of this research was its short-term follow up. It would be interesting to determinate the duration of improvement for all the outcomes. Actual osteopathic practice usually applies a combination of multiple techniques, during several sessions. However, our research was limited to one technique for two sessions, because we wanted to know the specific effects of a single visceral osteopathic technique, without the influence of other manual procedures. A more comprehensive osteopathic treatment might achieve even better results. Furthermore, despite the absence of statistical significance, baseline GerdQ differences must be considered to analyze the results. The absence of practitioner blinding must be taken into account, as well as the fact that whether participant blinding was convincing or not was not checked.

We have obtained 37.8% of improvement in GerdQ scores (medium effect size) after one week, by means of the application of a single session lasting 5 min. To interpret these results, the fact that treatment was quite short, as was the follow-up, baseline scores were low, and adverse events were very scarce, must be taken into consideration. Martinez–Hurtado et al. [21] obtained 73.9% of improvement at one week, through the application of two 25 min sessions of a myofascial release protocol, composed of six different techniques. However, they did not use the same questionnaire to evaluate GERD symptoms, and their treatment was much longer than ours.

Considering the same questionnaire (GerdQ) in the respective languages, at 4 weeks, Shih et al. [54] obtained a 73.1% improvement (scores improved from 4.92 to 1.32) in the omeprazole (one capsule per day) group and 65.5% (from 6.15 to 2.13) in the phytotherapy (three intakes per day) group. On the other hand, Rimmani et al. [55] got a 34.7% improvement (from 10 to 6.47) after one week in the questionnaire scores, using dexlansoprazole (one tablet per day) during Ramadan. Yu et al. [56] achieved, after 2 weeks, a 31.57% improvement (from 10.58 to 7.24) with one daily pill of esomeprazole, plus two daily pills of flupentixol/melitracen, and 36.79% (from 10.98 to 6.94), with one pill of esomeprazole per day.

With respect to other studies with a one week follow-up, using other questionnaires to evaluate GERD symptoms, Reimer et al. [57] got a 52.63% improvement with alginate (taken four times a day) plus a daily PPI, while the placebo plus daily PPI group got a 33.33% improvement, measured by the Heartburn Reflux Dyspepsia Questionnaire. Cossentino et al. [58], using a standard questionnaire, obtained a 46.42% improvement with a GABA_B_ agonist (daily intake of varied doses), while the placebo group achieved a 28.57% improvement.

Despite other interventions seeming to achieve a higher rate of improvement than us, our results confirm previous preliminary results about the usefulness of manual interventions for GERD [21]. Hence, osteopathic manual intervention could be an interesting alternative to drugs for patients with multiple medications, which require prioritizing some over others to avoid interaction. Recent studies consider three main reasons for developing GERD [6,59]. Esophageal shortening during transient lower sphincter relaxation is one of these reasons. The extension of the whole spine of the patient, combined with the caudal push executed by the osteopath during the intervention in the EG, might facilitate esophageal elongation, and explain the improvement in GERD symptoms. However, the elongation of the esophagus was not measured, so this cannot be established.

Another factor described as causing GERD is the transient lower sphincter relaxation. A previous study showed that osteopathic manual treatment for the diaphragm achieved better results to increase lower esophageal sphincter pressure than a sham maneuver [22]. The technique that we have used in our trial has several aspects in common with that study, since both maneuvers promote a long excursion of the diaphragm, deep breathing and rib mobilization. So, in our study, an increase in lower esophageal sphincter pressure might be expected.

The third main cause for GERD development is the pressure gradient across the esophagogastric junction during transient lower sphincter relaxation, if the stomach presents higher pressure than esophagus. In this sense, kyphotic posture has been shown to increase intraabdominal pressure [60,61]. In our study, a lordotic mobilization was applied, since the patient was asked to straighten his whole spine while breathing out, and both components of the exercise might decrease intraabdominal pressure, so this fact might have helped modify the pressure gradient and improve GERD symptoms.

On the other hand, the effect of the treatment in our study might only be due to the effects of mobilization. The movement of visceral structures is a physiological issue, and it is known that movement is beneficial in order to properly develop visceral functions [62,63,64]. It must also be taken into account that the esophagus, the sphincter and the diaphragm are constituted of myofascial tissues, and it is known that kind movement, pressure and stretching tend to improve the state of myofascial tissues in general terms. These three factors (kind mobilization, pressure and stretching of the esophagus, sphincter and diaphragm) might occur during the application of the osteopathic manual technique, and all of them can have a degree of influence in restoring normal muscle contraction/relaxation to the crural diaphragm, thus its influence on the gastroesophageal junction in the pathology of reflux. Besides the mechanical effects, it has been proposed that osteopathy and other manual medicines might achieve their goals by means of interoceptive effects [65] or by means of skin stimulation [66]. However, although the CG also received skin contact and moved in the same way, the EG achieved greater improvements.

Regarding PPT, in our study, C4 sensitivity was tested due to the indispensable participation of this cervical root in the composition of the phrenic nerve, which innervates the diaphragm and other upper abdominal structures [33,67]. The improvement of these structures, innervated by the phrenic nerve, might produce a reduction in cervical tissues’ sensitivity, as happened in our study. This relation has been previously considered in the chronic nonspecific neck pain population suffering from dyspepsia [34]. These authors found that a single osteopathic mobilization of the stomach and liver reduced neck pain and improved the upper trapezius electromyographic activity immediately and 7 days after treatment. In fact, it had already been shown that osteopathic visceral treatment was able to diminish sensitization in spinous processes of vertebrae related to sympathetic innervation of the colon in patients with constipation [20]. The same hypoalgesic effect has been found in asymptomatic subjects in the tissues which share innervation with the manually treated structure: diaphragm–C4 [68] and colon–L1 [69]. So, it seems that manual intervention focused on upper abdominal visceral disorders diminishes cervical sensitization, besides improving GerdQ scores. This is an important issue, considering that (i) gastroesophageal reflux [70,71] and chronic neck pain [72,73,74] are both related to hypersensitivity, (ii) neck pain is closely related to digestive disorders [75], and (iii) pain referred from the stomach and diaphragm is perceived in C4 dermatome [29,76].

In reference to cervical mobility, in our study, range of motion did not improve after the first intervention, but improved after the second. This result for the first treatment is similar to those obtained in subjects with dyspepsia [34], but they did not apply a second treatment. These results might mean that a more comprehensive treatment is needed to achieve an improvement in cervical range of motion. The improvement in cervical mobility might be explained by the improvement of GERD, diaphragm and lower esophageal sphincter, and the subsequent reduction in hypersensitivity and triggering. Previous studies have shown that cervical tightness and spasm diminish and disappear after medical and/or surgical treatment for GERD [27,28,30]. This muscular relaxation effect might need more time to be obtained [28].

On a practical level, this research helps increase knowledge of the visceral effects of manual interventions, and to specifically consider manual therapy, and this technique, as an option for GERD treatment. However, more research is needed to understand the role of osteopathy in the multidisciplinary management of GERD. Meanwhile, our results show that the population that most benefits from the osteopathic technique for GERD symptoms are those with lower severity of symptoms. However, this implication was not found for cervical sensitivity or mobility. In respect to the somatic effects of this technique, this study supports its application to improve neck pressure pain thresholds and mobility, when they are affected due to GERD, or the affectation is concurrent. Furthermore, our results support the relation between the severity of visceral disorders and somatic hypersensitivity, pointing out the need to make a differential diagnosis to evaluate if neck sensitization is due to GERD.

## 5. Conclusions

In view of these findings, osteopathic visceral technique for GERD improves GERD symptomatology, C4 spinous process PPT and cervical mobility. Further, a correlation has been found between greater GERD symptomatology and lower C4 spinous process PPT.

## Figures and Tables

**Table 1:** Baseline Characteristics of Participants.

Characteristics	Control Group (*n* = 29)		Osteopathic Manual Group (*n* = 31)		*p* Value
	Mean	SD (95%CI)	Mean	SD (95%CI)	
Age	49.45	13.77 (44.21–54.69)	48.19	14.03 (43.05–53.34)	0.728
Sex					
Male	14	48.3%	15	48.4%	0.599
Female	15	51.7%	16	51.6%	
PPIs					
Yes	8	27.6%	14	45.2%	0.188
No	21	72.4%	17	54.8%	
Smoker					
Yes	7	24.1%	12	38.7%	0.175
No	22	75.9%	19	61.3%	
BMI	24.81	3.84 (23.35–26.27)	24.70	3.84 (23.30–26.11)	0.706
PPT C4	30.49	8.44 (27.28–33.70)	29.93	12.63 (25.30–34.56)	0.304
Cervical mobility	328.65	62.22 (306.34–350.97)	326.16	57.91 (304.57–347.74)	0.873
GerdQ test	3.79	2.81 (2.73–4.86)	5.13	3.91 (3.69–6.56)	0.248

PPIs, Proton pump inhibitors; BMI, Body mass index; PPT, Pressure pain threshold; SD, standard deviation; CI, confidence interval.

**Table 2:** Outcome measures and statistical significance of the inter-group pairwise comparisons.

Outcome	Time	Control Group (*n* = 29)		Osteopathic Manual Group (*n* = 31)		*p* Value
		Mean	SD (95%CI)	Mean	SD (95%CI)	
PPT C4	Baseline	30.49	8.44 (26.47–34.51)	29.92	12.63 (26.04–33.81)	0.034 ^a^
	Post-1st Treatment	29.25	9.48 (25.09–33.41)	29.36	12.57 (25.34–33.39)	
	Follow-up	29.31	11.25 (23.15–35.46)	34.06	20.28 (28.05–39.95)	
	Post-2nd Treatment	29.61	11.14 (22.83–36.4)	37.84	22.97 (31.28–44.40)	
Cervical mobility	Baseline	328.65	62.22 (306.34–350.97)	326.16	57.91 (304.57–347.74)	<0.001 ^a^
	Post-1st Treatment	319.55	60.56 (299.20–339.90)	339.51	48.69 (319.83–359.19)	
	Follow-up	309.20	59.96 (288.46–329.94)	336.96	51.58 (316.91–357.02)	
	Post-2nd Treatment	312.86	64.72 (291.56–3334.15)	344.25	49.33 (323.66–364.85)	
GerdQ test	Baseline	3.79	2.81 (2.73 to 4.86)	5.13	3.91 (3.69–6.56)	0.005 ^b^
	Post 1 Week	3.34	2.81 (2.27–4.42)	3.19	3.37 (1.96–4.43)	

^a^*p* value: results of test of within-subjects effects (based on Sphericity Assumed). ^b^ *p* value: based on T Student test results. PPT, Pressure pain threshold; SD, standard deviation; CI, confidence interval; CG, control group; OMG, osteopathic manual group; Post-1st Treatment, after first intervention; Follow-up, after a week; Post-2nd Treatment, after second intervention.

**Table 3:** Absolute between-group differences.

Outcome	Time	Mean	95% CI
GerdQ test	Baseline	1.34	−0.42–3.09
	Post 1 Week	0.15	−1.46–1.763
	Difference Post 1 Week - Baseline	1.49	0.47–2.49
PPT	Baseline	0.56	−5.03–6.15
	Post-1st Treatment	0.11	−5.67–5.90
	Follow-up	4.69	−3.75–13.14
	Post-2nd Treatment	8.22	−1.08–17.53
	Difference Post 2nd Treatment - Baseline	8.78	0.48–17.09
Cervical mobility	Baseline	2.49	−28.55–33.54
	Post-1st Treatment	19.96	−8.34–48.27
	Follow-up	27.76	−1.09–56.61
	Post-2nd Treatment	31.39	1.77–61.02
	Difference Post 2nd Treatment - Baseline	33.89	15.17–52.61

PPT, Pressure pain threshold; CI, confidence interval; Post-1st Treatment, after first intervention; Follow-up, after a week; Post-2nd Treatment, after second intervention.
